# Phenotypic divergence of *Glossina morsitans* (Diptera: Glossinidae) populations in Zambia: Application of landmark‐based wing geometric morphometrics to discriminate population‐level variation

**DOI:** 10.1002/ece3.70348

**Published:** 2024-09-30

**Authors:** Jackson Muyobela, Christian W. W. Pirk, Abdullahi A. Yusuf, Catherine L. Sole

**Affiliations:** ^1^ Department of Zoology and Entomology University of Pretoria Hatfield Pretoria South Africa; ^2^ Department of Veterinary Services, Tsetse and Trypanosomiasis Control Unit Ministry of Fisheries and Livestock Lusaka Zambia

**Keywords:** adaptation, centroid size, divergence, population structure, wing shape

## Abstract

An important consequence of the discontinuous distribution of insect populations within their geographic range is phenotypic divergence. Detection of this divergence can be challenging when it occurs through subtle shifts in morphological traits with complex geometries, such as insect wing venation. Here, we used landmark‐based wing geometric morphometrics to investigate the population‐level phenotypic variation of the two subspecies of *Glossina morsitans*, *G. m. centralis* Machado and *G. m. morsitans* Westwood that occur in Zambia. Twelve homologous landmarks digitised on the right wings of 720 specimens collected from four and five sites (80 per site with 1:1 sex ratio) within the *G. m. centralis* and *G. m. morsitans* range respectively, were subjected to generalised Procrustes analysis to obtain wing centroid size (CS) and wing shape variables. Linear permutation models and redundancy analysis were then used to compare CS and wing shape between male and female *G. morsitans*, the two subspecies *G. m. centralis* and *G. m. morsitans*, the sexes of each subspecies and between sample locations within each subspecies range, respectively. Significant differences in CS and wing shape were observed between *G. morsitans* sexes, subspecies and sample locations within each subspecies range. A neighbour‐joining cladogram derived from the analysis of Procrustes distances showed that tsetse within each subspecies range were highly divergent. We conclude that *G. morsitans* populations in Zambia exhibit significant population‐level variation in fly size and wing shape which suggests high levels of population structuring. The main drivers of this structuring could be random genetic drift in *G. m. centralis* demes and local adaptation to environmental conditions in *G. m. morsitans* populations. We therefore recommend molecular studies to estimate the levels of gene flow between these populations and identify possible barriers to genetic flow.

## Graphical abstract

Land‐mark based geomorphometric wing analysis of Glossina morsitans populations in Zambia shows that this tsetse exhibit significant population‐level variation in fly size and wing shape suggesting high levels of population structuring. The main drivers of this structuring could be random genetic drift and local adaptation to environmental conditions.

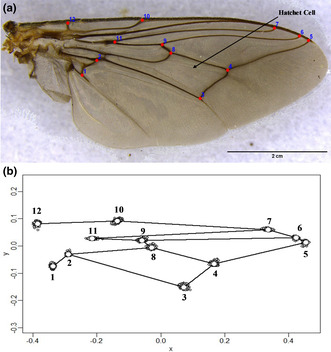

## 1 INTRODUCTION

*Glossina morsitans* (Diptera: Glossinidae) is a savannah tsetse species of the subgenus *Glossina* (*morsitans* group) whose distribution is restricted to savannah woodlands (Leak et al., 2008) and is correlated with that of wildlife (Vreysen et al., 2013). Three allopatric subspecies occur, namely, *G. m. submorsitans* Newstead, *G. m. centralis* Machado, and *G. m. morsitans* Westwood (Jordan, 1993), all of which are efficient vectors of trypanosomes (Kinetoplastida: Trypanosomatidae), which cause human and animal trypanosomiasis in sub‐Saharan Africa (Rogers, 2000). The geographical distribution of *G. m. submorsitans* is from Western to Central Africa, while *G. m. centralis* and *G. m. morsitans* occur in Eastern, Central, and Southern Africa (Rogers & Robinson, 2004). In Zambia, *G. m. centralis* and *G. m. morsitans* are predicted to occupy 151,353 km^2^ or 20% of the land mass (Muyobela et al., 2023).

In conformity with most insect species, the distribution of *G. morsitans* within its geographic range is generally discontinuous (Krafsur, 2009; Muyobela et al., 2023), being strategically arranged based on the availability of food sources, reproductive needs, dispersal capacity, and local environmental conditions (Dujardin, 2008). The spatial arrangement of a species based on environmental heterogeneity can lead to divergent selection whereby local population demes evolve traits that provide an advantage under local environmental conditions regardless of the consequences for fitness in other habitats (Williams, 1966). In the presence of restricted gene flow (due to passive dispersal or active habitat selection), strong selection against genotypes adapted to other habitats, moderate selection against intermediate genotypes, little temporal variation in forces of selection, and small differences in habitat size and quality (e.g. resource availability), such population demes become locally adapted (Kawecki & Ebert, 2004). Local adaptation can give rise to population‐level phenotypic variation that may result in the structuring of populations into biogeographical islands or subpopulations (Dujardin & Le Pont, 2004; Getahun et al., 2014; Mbewe et al., 2018). Where significant barriers to gene flow exist, these subpopulations become isolated and undergo rapid evolutionary changes in morphological traits due to founder effects and genetic drift (Ostwald et al., 2023). The identification of isolated tsetse populations has been deemed crucial for the successful and sustainable implementation of area‐wide integrated vector management (AW‐IVM) (Bouyer et al., 2010; Kgori et al., 2006), guiding the decision whether to undertake suppression or elimination campaigns (Bouyer et al., 2007).

A relatively low‐cost approach for investigating tsetse population structure is the use of landmark‐based geometric morphometrics (GM), defined as the statistical analysis of shape variation and its covariation with other variables (Rohlf & Bookstein, 2003). Unlike traditional morphometrics, GM is a powerful technique that captures the geometry of the morphological structure under study and retains this information throughout the analysis (Zelditch et al., 2004). The procedure is accomplished through the Procrustes paradigm (Adams et al., 2013) in which a set of two‐dimensional landmark coordinates recording the relative positions of homologous anatomical points are obtained and then subjected to generalised procrustes analysis (GPA) (Rohlf & Slice, 1990). This least‐squares superimposition technique produces a set of shape variables whose geometric dissimilarity is expressed as the Procrustes distance between the homologous points of two configurations (Zelditch et al., 2004) and whose pattern of variation can be visualised by graphical methods (Baken et al., 2021). An additional output of this analysis is centroid size (CS), defined as the square root of the summed squared distance of each landmark from the centroid of the form (Tatsuta et al., 2018). This isometric measure of size is used as an estimator of the global size of the form under study in GM studies (Dujardin, 2008).

Conspecific size variability within and among insect populations is generally known to be an environmentally induced and reversible character (Jirakanjanakit et al., 2007). In *G. morsitans*, size variability has been attributed to seasonal effects (Hargrove et al., 2019) with temperature being the major source of variation (Glasgow, 1961; Phelps & Clarke, 1974). High heritability values for insect size have however been reported (Lehmann et al., 2006) and the transgenerational effects of size among the *Glossina spp* have been demonstrated (Mbewe et al., 2018). Therefore, heritable size variation can be used to discriminate populations. Size‐corrected or allometry‐free shape is known to be a polygenic character and strong evidence of its genetic determinism has been provided (Klingenberg & Leamy, 2001; Patterson & Klingenberg, 2007). Allometry‐free shape has also been shown to be a powerful discriminator of groups (Dujardin, 2008) and is, therefore, a very useful tool in taxonomic studies (Klingenberg, 2016).

The insect body part most subjected to GM studies is the wing (Tatsuta et al., 2018). This is due to several reasons. Firstly, insect wings are almost entirely two‐dimensional structures, a fact that greatly reduces digitisation errors (Dujardin, 2008). Secondly, the arrangement and branching patterns of insect wing veins contain taxonomic information that has been used to construct classification schemes, infer phylogeny (Bybee et al., 2008), elucidate evolutionary patterns (Debat et al., 2003), and evaluate fluctuating asymmetry – deviations from perfect symmetry that indicate developmental noise (Klingenberg et al., 2001). Lastly, the geometric shape of insect wings has been shown to exhibit high environmental canalisation – the ability of a genotype's phenotype to remain relatively invariant when exposed to different environments (Henry et al., 2010). These attributes, therefore, make the geometric shape of insect wings, a suitable phenotypic character to distinguish conspecific populations and species using GM (Dujardin, 2011). Insect wing shape is captured by placing homologous landmarks on the intersection of wing veins.

Geometric morphometrics has been used to study natural population variation in several insect species including the common fruit fly *Drosophila* (Diptera: Drosophilidae) (Gilchrist et al., 2000), honey bee *Apis* (Hymenoptera: Apidae) (Radloff & Hepburn, 2000), sand fly *Lutzomyia* (Diptera: Psychididae) (Dujardin & Le Pont, 2004), triatomine bug *Rhodnius* (Hemiptera: Reduviidae) (Villegas et al., 2002) and culicid mosquitoes *Culex*, *Aedes* and *Anopheles* (Diptera: Culicidae) (Virginio et al., 2015). Among the *Glossina* geometric morphometrics has been used to study phenetic variation in *G. palpalis gambiensis* (Bouyer et al., 2007; Solano et al., 1999), *G. p. palpalis* (Ebhodaghe et al., 2017; Kaba et al., 2012), *G. m. submorsitans* (Achukwi et al., 2013), *G. pallidipes* (Getahun et al., 2014), *G. austeni* (De Beer et al., 2019), *G. fuscipes fuscipes* (Mbewe et al., 2018), *G. tachinoides* (Mustapha et al., 2018) and *G. brevipalpis* (De Beer et al., 2019). However, phenotypic variation in natural populations of *G. m. centralis* and *G. m. morsitans* has not been investigated. Therefore, this study aimed to use landmark‐based wing geometric morphometrics to investigate phenotypic variation and determine the level of population structuring in *G. m. centralis* and *G. m. morsitans* populations in Zambia.

## 2 MATERIALS AND METHODS

### 2.1 Study sites

**FIGURE 1:**
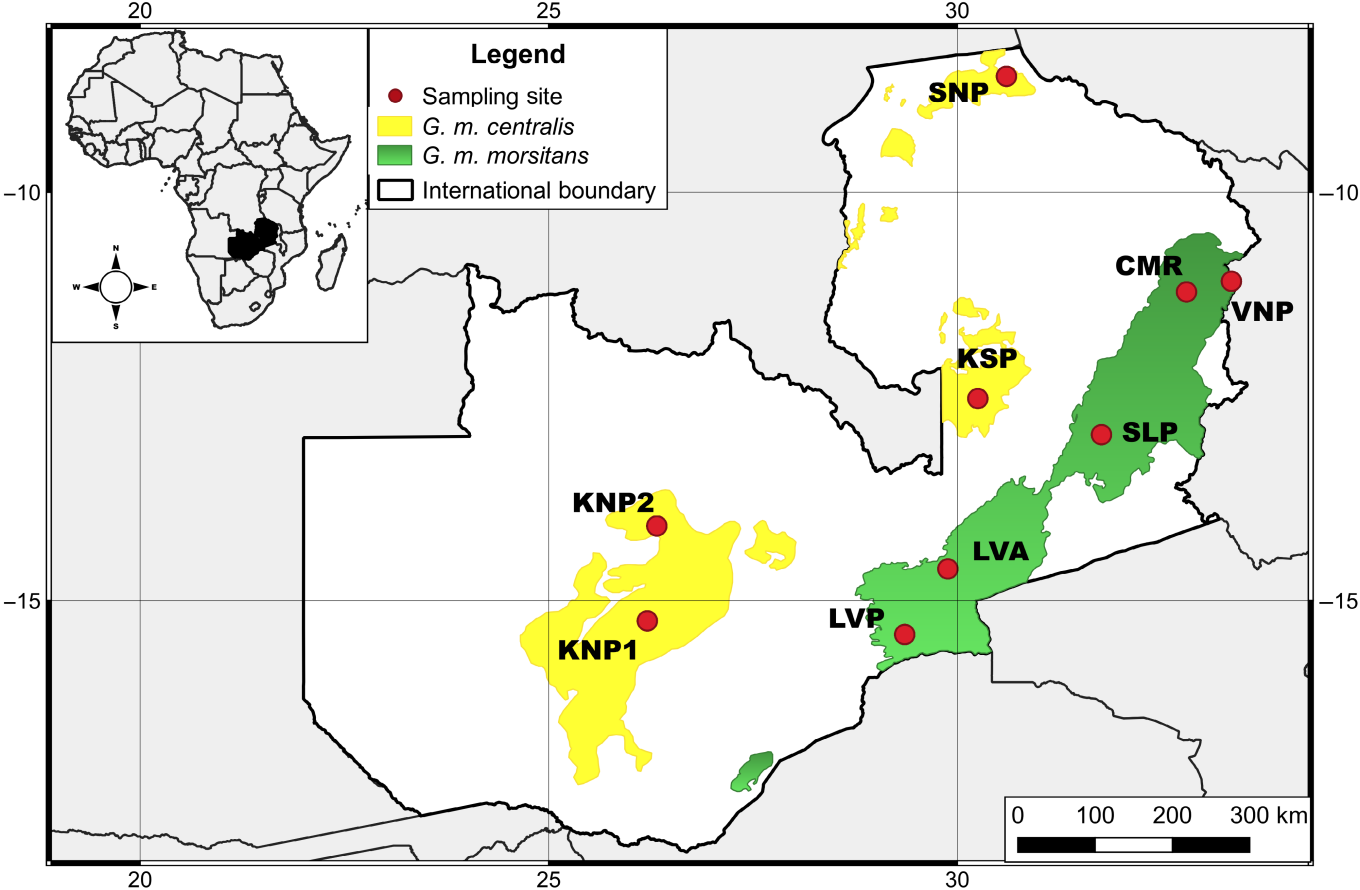
Distribution of *G. m. centralis* and *G. m. morsitans* in Zambia. Data on each subspecies in Muyobela et al. (2023). The base map layer was obtained from the Database of Global Administrative Area GADM (https://geodata.ucdavis.edu/gadm/gadm4.1/shp/gadm41_ZMB_shp.zip) and under the licence https://gadm.org/license.html. The figure was created using QGISv3.0 (http://qgis.org/en/site/).

The study was carried out in Zambia, between the longitudes 22 and 34°E, and latitudes 8 and 18°S. The two *G. morsitans* subspecies exhibit an allopatric distribution with *G. m. morsitans* occupying the hotter Eastern part and the other subspecies, *G. m. centralis*, occupying the cooler Western and Northern part of the country (Figure 1). The habitat of *G. m. centralis* is characterised by Miombo woodland interspaced with large dambos (grassy wetlands) with high annual rainfall (above 1000 mm) (Wigg, 1949). Mopane woodland is the dominant vegetation in the *G. m. morsitans* range with moderate to low annual rainfall (<800 mm). *Glossina m. centralis* was collected from four sites, namely Mumbwa South (KNP1) and Kasongo Busanga (KNP2) game management areas, and Kasanka (KSP) and Sumbu (SNP) national parks (Figure 1), while *G. m. morsitans* was captured in five sites: Mulangu (CMR and VNP) and Luano (LVA) game management areas, and South Luangwa (SLP) and Lower Zambezi (LZP) national parks (Figure 1).

### 2.2 Tsetse samples

The data used in this study form a subset of results of a cross‐sectional tsetse survey conducted between September 2021 and August 2022 (Muyobela et al., 2023). The subset consists of flies captured in November 2021, chosen because this was the only month that recorded catches in all sample sites. The sampling was done using the vehicle‐mounted sticky trap (VST) (Muyobela et al., 2021) baited with butanone and 1‐octen‐3‐ol dispensed at a rate of 150 and 0.5 mg/h. respectively (Torr et al., 1997). Tsetse captured within a 2‐km radius of a sample site were amalgamated from which 80 non‐teneral (40 males and 40 females) flies with intact wings were selected. Subspecies identity was confirmed by dissecting male genitalia (hypopygium) as described by Leak et al. (2008). *Glossina m. morsitans* subspecies was identified by the presence of narrow median lobes on superior claspers of the hypopygium that had slightly divergent tips. The median lobes of *G. m. centralis* were relatively wider, with tips markedly divergent. A total of 720 (360 *G. m. centralis* and *G. m. morsitans*) were used in the study.

### 2.3 Wing measurements and Procrustes superimposition

**FIGURE 2:**
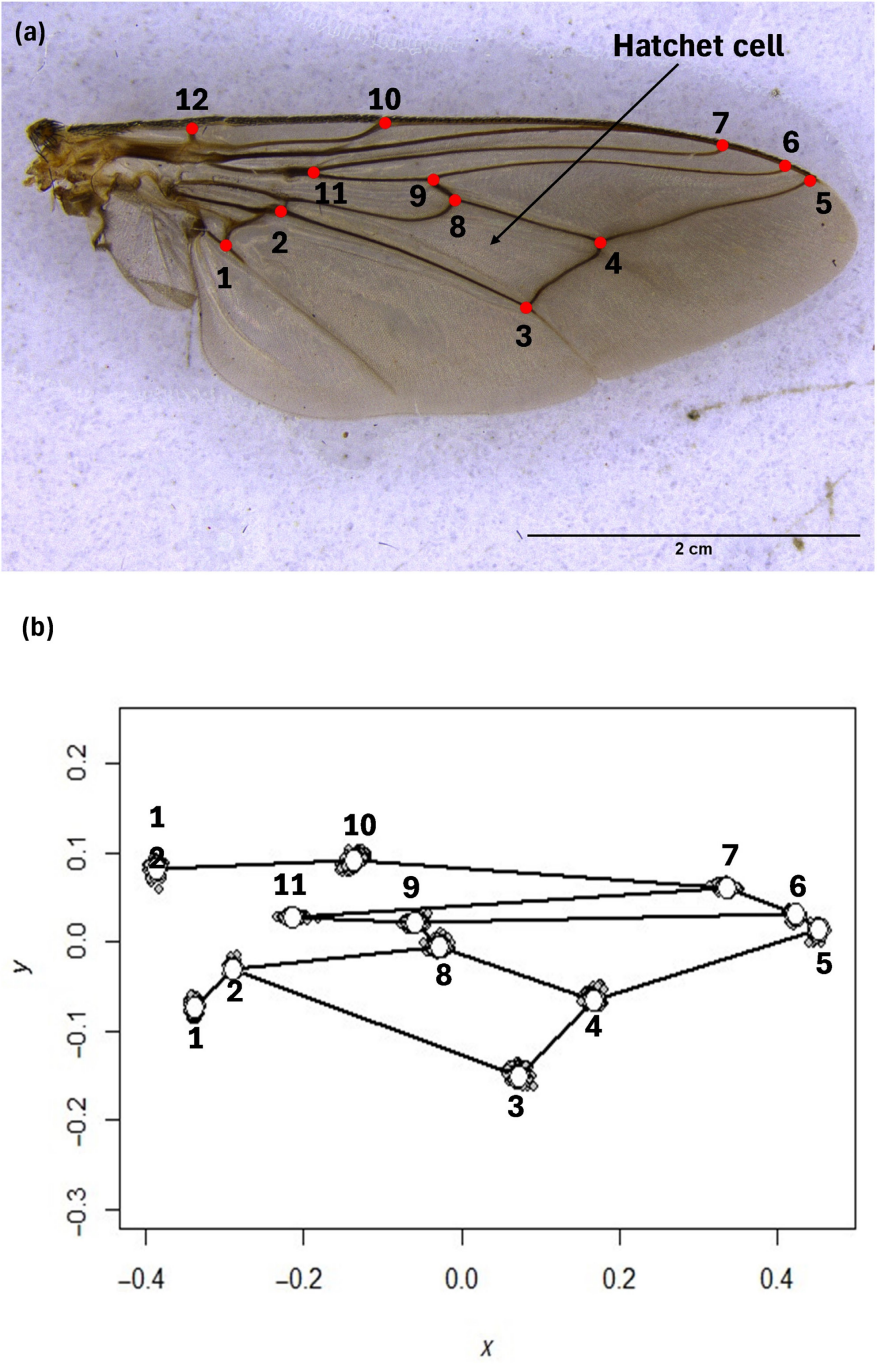
Landmark digitisation and general Procrustes analysis. (a) Image of the 12 landmarks and the order of landmark collection from the right wing of *G*. *morsitans*. (b) Scatter plot with wireframe links of landmark configurations of all 720 wings in the dataset after Procrustes superimposition. For each landmark, the white circle indicates the location of the landmark for the average shape and the grey dots indicate the locations for individual wings.

The right wing of each fly was mounted on a glass slide and affixed with transparent sticky tape. The wings were then photographed using a Leica M165C stereomicroscope attached to a Leica camera (DMC‐2900) (Leica Microsystems, Germany). The images were compiled using tpsUtil v1.79 (Rohlf, 2015) and digitised with tpsDig2 v2.32 (Rohlf, 2015). Twelve homologous landmarks defined as junctions of wing veins were identified and digitised (Figure 2a). To avoid individual bias, landmark digitisation was undertaken by the same person. To avoid operational bias during digitization, specimens were selected at random.

Procrustes superimposition of landmark configurations was performed using general Procrustes analysis (GPA) using Geomorph version 4.0 package (Baken et al., 2021) in R (R Core Development Team, 2015). The procedure translated all landmark configurations to a common location, scaled them to unit CS, and rotated them into an optimal least‐squares alignment with an iteratively estimated mean reference form (Zelditch et al., 2004) so that the sum of squared distances between corresponding landmarks of each configuration and the mean configuration was minimised (Klingenberg, 2013). This analysis produced the Procrustes distances which measure shape dissimilarity as well as the CS. A scatter plot of superimposed landmarks for all specimens is shown in Figure 2b.

**FIGURE 3:**
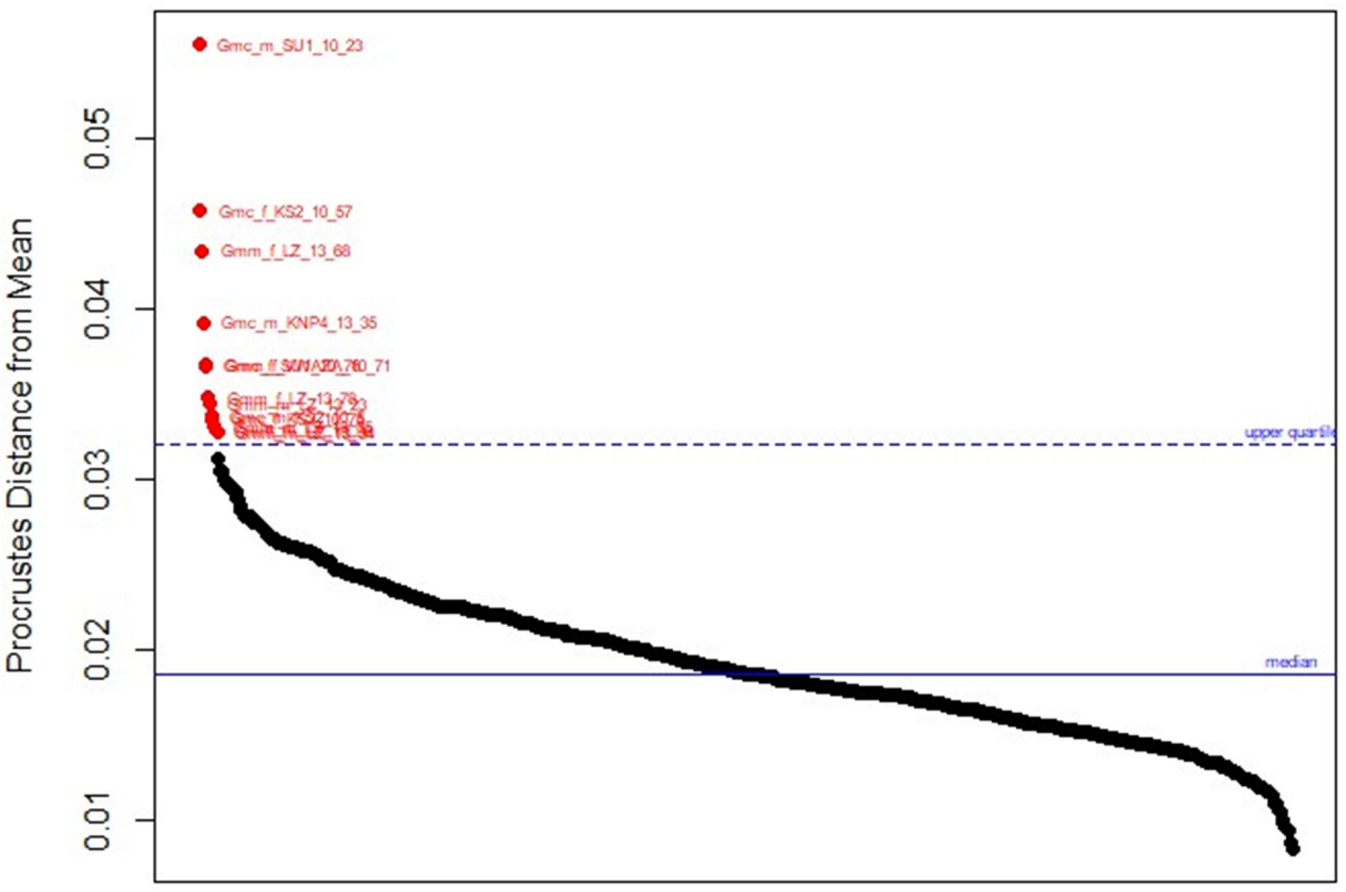
Procrustes distance of each specimen from the mean shape. The plot shows that specimen Gmc_m_SU1_10_23 had the largest distance from the mean shape and was therefore considered to be an outlier.

Digitisation errors were identified by plotting the ordered Procrustes distance of aligned specimens from the mean shape (Sherratt, 2016) (Figure 3) using the Geomorph package in R. Specimens that have been digitised wrongly (for example, mixing up the order of landmarks) exhibit large variances and therefore fall outside the upper quartile range of the plot. As shown in Figure 3, the specimen Gmc_m_SU1_10_23 was observed to be furthest from the upper quartile range of the plot and was therefore identified as an outlier. This specimen was therefore omitted from further analysis.

**FIGURE 4:**
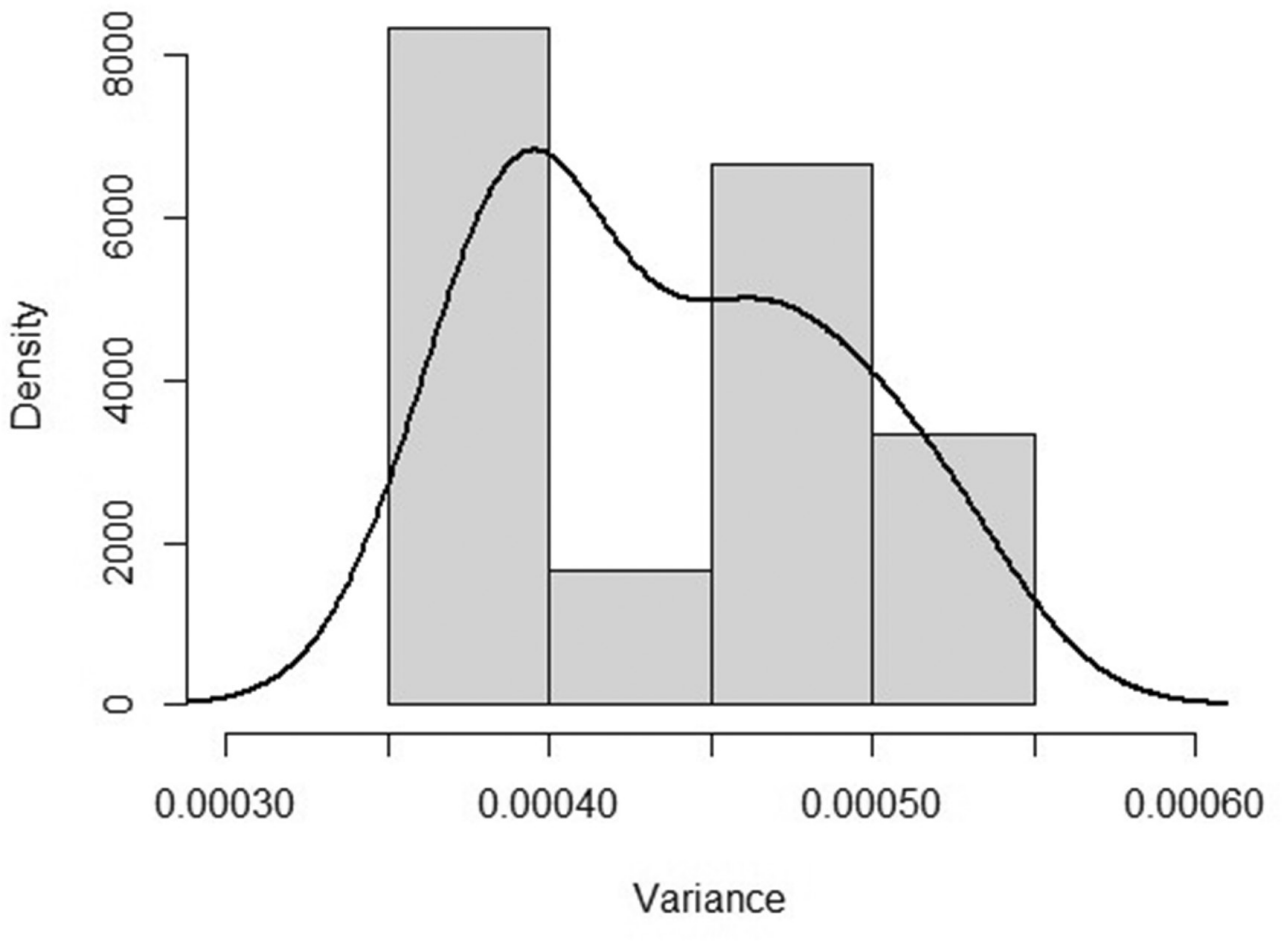
Distribution of landmark variance. The histogram indicates that all landmarks are part of the same distribution, and no outlier is present in the dataset.

The ability to reliably locate and digitise landmarks was determined by assessing the variance contribution of each landmark to the mean shape since landmark locations are not independent quantities but are relative to all other landmarks (Zelditch et al., 2004). This was done by sequentially computing the variation in landmark position around the mean shape, omitting one landmark each time the computation was made (Sheets, 2014). Omitting a landmark that is difficult to reliably digitise results in a decrease in variance around the mean, relative to the variation seen when other landmarks are omitted. This jackknife computation of variance was done in CoordGen8 (Sheets, 2014). As shown in Table 1, landmark 10 was found to be the most difficult to reliably locate and digitise. However, a histogram plot of variance density (Figure 4) showed that the variance of landmark 10 was part of a smooth distribution of variance around landmarks. Landmark 10 was therefore included in the study.

**TABLE 1:** The variance around the mean shape as each landmark is omitted in turn.

LM omitted	Variance
10	0.000368
3	0.000389
11	0.000391
8	0.000394
9	0.000396
4	0.000404
1	0.00045
7	0.000452
2	0.000476
12	0.000476
6	0.000511
5	0.000524

*Note*: Low variance when a landmark is excluded indicates that the landmark contributes greatly to the total variance.

### 2.4 Environmental data and processing

Elevation, annual temperature, isothermality, annual precipitation, land surface temperature, and vegetation cover are among the most important variables affecting the biology of *Glossina spp* (Challier, 1982; Muyobela et al., 2023; Nnko et al., 2021). Therefore, these variables were selected to assess the spatial environmental heterogeneity of sample sites and to estimate their effect on phenotypic variation. Annual temperature, isothermality, and annual precipitation data were obtained from WorldClim Global Climate Database version 2.1 (Fick & Hijmans, 2017). Moderate Resolution Imaging Spectroradiometer (MODIS) composite time series land surface temperature day (LST) (MOD11A1) (Didan, 2015) and per cent tree cover based on the Vegetation Continuous Fields (VCF) (MOD44B) (DiMiceli et al., 2015) were obtained from NASA's EOSDIS Land Processes Distributed Active Archive Center (AppEEARS Team, 2022). Elevation data was obtained as Global 30 Arc‐Second Elevation (GTOPO30) from the Earth Resources Observation and Science Center (Earth Resources Observation and Science Center/U.S. Geological Survey/U.S. Department of the Interior, 1997).

Harmonic regression was performed on monthly time series LST data using the TSA package (Kung‐Sik & Ripley, 2020) in R (R Core Development Team, 2015). The first coefficient in the regression, representing the mean of the variable, was selected for further analysis. Data values for all environmental variables at each sampling site were extracted using the Raster package (Hijmans & van Etten, 2012) in R.

### 2.5 Data analyses

#### 2.5.1 Spatial autocorrelation analysis

Spatial autocorrelation is the positive or negative correlation of a variable with itself due to the spatial location of observations (Salima & de Bellefon, 2018). Residues of statistical models based on spatially autocorrelated data violate the key assumption of standard statistical tests, that residues are independent and identically distributed (Dormann et al., 2007). Violation of this assumption may bias parameter estimates and increase Type I error rates (falsely rejecting the null hypothesis of no effect). To ensure statistical independence of CS and shape variables, Global Spatial Autocorrelation Tests were conducted. For CS, a permutation Moran's *I* test was used to assess the strength of spatial autocorrelation using the spdep package (Bivand & Wong, 2018) in R for both *G. m. centralis* and *G. m. morsitans*. Mantel (Mantel, 1967) and Partial Mantel (Guillot & Rousset, 2013) tests were used to evaluate spatial autocorrelation of shape and environmental variables for both *G. m. centralis* and *G. m. morsitans* using the EcoGenetics package (Roser et al., 2017) in R.

#### 2.5.2 Environmental characterisation of sample sites

Linear permutation models with 2000 iterations were used to test for the differences in elevation, annual temperature, isothermality, annual precipitation, land surface temperature, and per cent tree cover between *G. m. centralis* and *G. m. morsitans* sample sites using the Geomorph package in R. Principal components analysis (PCA) was used for the multivariate analysis of these environmental variables to identify the most important variables accounting for environmental variability between sample sites.

#### 2.5.3 CS analysis

Shapiro–Wilk normality test showed that both CS and log CS were not normally distributed (*p = .001* for both variables). Therefore, permutation procedures were used to analyse CS. Linear permutation models with 2000 iterations were used to compare wing CS differences between *G. morsitans* males and females, *G. m. centralis*, and *G. m. morsitans* subspecies, males and females of each subspecies, as well as CS differences between sample locations within each subspecies range using the Geomorph package in R. The pairwise function was used for multiple group comparisons where CS was observed to be different between sample locations. Linear permutation models were further used to estimate the effect of elevation, annual temperature, isothermality, annual precipitation, land surface temperature, and per cent tree cover on wing CS.

#### 2.5.4 Allometric test and construction of allometry‐free shape variables

To test whether there was significant covariance between wing shape and size (allometry), multivariate linear permutation regression of wing shape on CS was conducted using the Geomorph package in R. Hypothesis testing was accomplished using Goodall's *F*‐test (Goodall, 1991), a statistical approach that partitions the variance of Procrustes distances rather than landmark coordinates. Goodall's *F*‐statistic is the ratio of explained (between‐group) and unexplained (within‐group) components of shape variation (Klingenberg, 2016) and has been demonstrated to have higher statistical power than other approaches (Rohlf, 2000). Residues from this regression were then used to construct allometry‐free shape variables that are recommended in taxonomic investigations (Klingenberg, 2009) and studies that define geographically constrained situations such as islands (Dujardin, 2011). The multivariate regression approach to remove the allometric component of shape variation offers a logical method as it partitions the variation in the dependent variables into predicted and residual components (Klingenberg, 2016). The predicted component corresponds to allometric variation of shape, whereas the residual component encompasses non‐allometric variation as residues are uncorrelated with CS.

#### 2.5.5 Shape analysis

Redundancy analysis (RDA) (Zuur et al., 2007) was used to model allometry‐free wing shape as a function of *G. morsitans* sex, subspecies, and geographic origin, using the vegan package (Oksanen et al., 2018) in R. The analysis consisted of the following steps. A multivariate linear permutation regression model was fitted to determine if *G. morsitans* allometry‐free wing shape variation was significantly influenced by sex differences, subspecies identity, and the two‐way interaction of these factors. The effect of geographic origin on allometry‐free shape variation in both *G. m. centralis* and *G. m. morsitans* was evaluated using multivariate linear permutation regression models, accounting for sex differences and the two‐way interaction between sex and geographic origin. Two PCAs were then performed on each regression model. A constrained PCA was applied to the fitted values of each regression model to summarise the variation in allometry‐free wing shape data that could be explained by the explanatory variables. An unconstrained PCA was then applied to the residues of the regression to estimate the variation not explained by these constraining variables. The total percentage of allometry‐free wing shape variation explained by sex and subspecies identity, and geographic origin within each subspecies range was estimated by the canonical *R* ^2^ bi‐multivariate redundancy statistic (Miller & Farr, 1971) calculated as proposed by Peres‐Neto et al. (2006) using the RVAideMemoire package (Hervé, 2023) in R. To test whether each variable explained a significant proportion of allometry‐free wing shape variation, a permutation *F*‐test based on the canonical *R* ^2^ (Legendre & Legendre, 2012) was used. Where differences between sample geographic origin were observed, multiple group comparisons were done using the RVAideMemoire package in R. Constrained PCA score plots were used to illustrate allometry‐free wing shape cluster separation due to sex, subspecies, and sample geographic origin within each subspecies range. To estimate the amount of shape variation that could be attributed to environmental variability, allometry‐free shape was regressed on elevation, annual temperature, isothermality, annual precipitation, land surface temperature and per cent tree cover with Goodall's *F*‐test used for hypothesis testing. A Procrustes distance matrix, computed from the fitted values of a multivariate linear permutation regression of *G. morsitans* allometry‐free shape variables on sex, subspecies and location, was used to build a neighbour‐joining cladogram to illustrate divergence of wing shape of flies from different locations.

#### 2.5.6 Isolation‐by‐distance test

Isolation‐by‐distance (IBD) hypothesis describes the pattern of population genetic variation that derives from spatially limited gene flow (Jensen et al., 2005) and is characterised by an increase in genetic or phenotypic differentiation among populations with increasing geographic distance (Van Strien et al., 2015). For IBD to occur, populations are assumed to be in gene‐flow‐drift equilibrium, experience no selection, and have dispersal rates that reduce with increasing geographic distance (Orsini et al., 2013). We evaluated whether allometry‐free wing shape variation among sample locations was due to IBD using the following procedures. Firstly, scatter plots were generated to visually assess the expected linear relationship between Procrustes and geographic distances under IBD for both *G. m. centralis* and *G. m. morsitans* populations. Secondly, Mantel‐based correlogram analysis (Roser et al., 2017) was used to statistically test the hypothesis of IBD in both subspecies ranges using the EcoGenetics package in R.

Alpha was set at .05 for all statistically significant analyses (Pirk et al., 2013).

### 2.6 Ethical statement

The protocol and procedures employed in this study were reviewed and approved by the Department of Zoology and Entomology at the University of Pretoria.

## 3 RESULTS

### 3.1 Spatial autocorrelation

Centroid size data for both *G. m. centralis* (Moran's *I* Statistic = 0.078, *p = .220*) and *G. m. morsitans* (Moran's *I* statistic = −0.270, *p = .595*) did not exhibit spatial autocorrelation. No spatial autocorrelation was observed among shape variables for both *G. m. centralis* (Mantel Statistic = −0.305, *p = .305*) and *G. m. morsitans* (Mantel Statistic = 0.089, *p = .344*). Environmental variables did not induce any spatial dependency in *G. m. centralis* (partial Mantel statistic = −0.315, *p = .318*) and *G. m. morsitans* (partial Mantel statistic = 0.451, *p = .344*) shape variables.

### 3.2 Sample site characterisation

**FIGURE 5:**
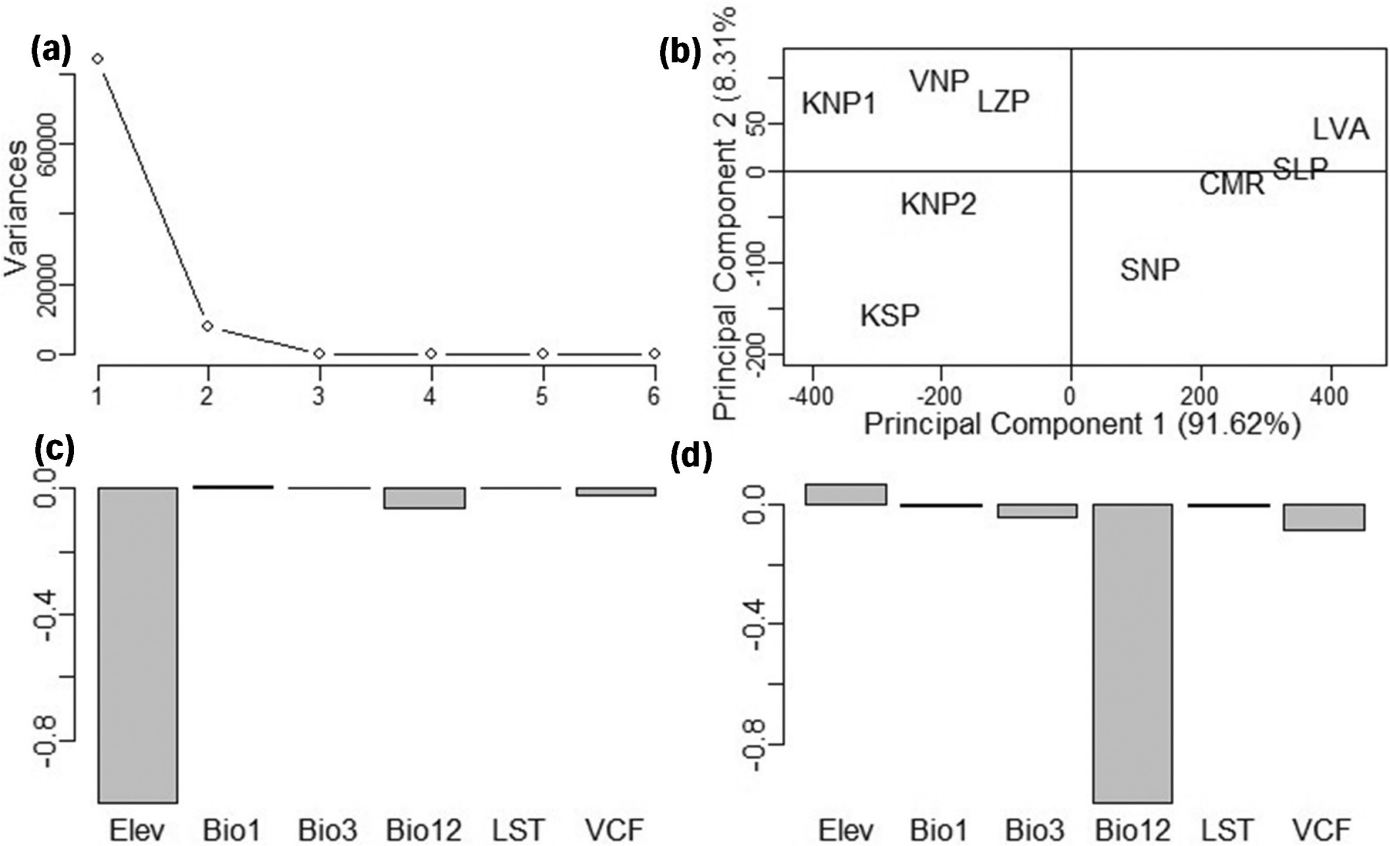
PCA of sample sites based on environmental variables. (a) Scree plot showing that most of the variance in the data set could be explained by the first two principal components PC1 and PC2. (b) Score plot indicating that PC1 and PC2 accounted for 91.62 and 8.31% of the variation among sites, respectively. (c) Vector loading plot showing that elevation and annual precipitation were the variables that contributed the highest variance to PC1. (d) Vector loading plot showing that annual precipitation, vegetation continuous field, and elevation contributed the highest variance to PC2. Bio1, Annual Temperature; Bio3, Isothermality; Elev, Elevation; LST, Land Surface Temperature; VCF, Vegetation Continuous Fields indicating per cent tree cover.

Elevation, isothermality, annual precipitation, and per cent tree cover were significantly lower in *G. m. morsitans* than in *G. m. centralis* sampling sites (*p < .001*). Annual temperature was observed to be higher in *G. m. morsitans* than in *G. m. centralis* range (*p < .001*). Land surface temperature was higher in four of the five sampling sites of *G. m. morsitans* than in those for *G. m. centralis* (*p < .001*). The LZP sample site for *G. m. morsitans* was observed to have LST 4°C lower than all other sampling sites. Within each subspecies range, environmental variables were observed to be significantly different between sample sites (*p < .001*). Elevation and annual precipitation were observed to be the environmental variables contributing most of the variation for principal component (PC) 1, whereas annual precipitation, vegetation continuous field (per cent tree cover) and elevation contributed the most for PC2 (Figure 5). PC 1 accounted for 91.62% of the variation between sites.

### 3.3 CS comparison

Significant wing CS differences were observed between male and female *G. morsitans* flies, the two subspecies *G. m. centralis* and *G. m. morsitans*, among male and female flies within each subspecies and between sample locations within the two subspecies ranges (Table 2). Male flies were observed to have an absolute size 9 per cent smaller than females and *G. m. morsitans* was 2 per cent smaller than *G. m. centralis*. At the subspecies level, male flies were observed to be 10 and 9 per cent smaller for *G. m. centralis* and *G. m. morsitans*, respectively. Within the *G. m. centralis* range, flies from KNP1 and KNP 2 were observed to be 3 per cent smaller than those from the KSP site (*p* < *.008* and *.013* respectively). In the *G. m. morsitans* range, flies from the LZP site were observed to be 5 per cent larger than flies from all other sites (*p* < *.001*).

**TABLE 2:** Sex, subspecies, and location comparison of mean wing CS in *Glossina morsitans*.

Experiment	Treatment	Mean CS (pixels)	Variance	Standard deviation (SD)	*p*‐Value
*G. morsitans*, male vs. females	Female	356	196.80	14.03	.001
	Male	323	89.37	9.45	
*G. m. centralis* vs. *G. m. morsitans*, subspecies	*G. m. centralis*	344	424.70	20.61	.001
	*G. m. morsitans*	336	383.41	19.58	
*G. m. centralis*, male vs. females	Female	363	103.36	10.17	.001
	Male	326	73.34	8.56	
*G. m. morsitans*, male vs. females	Female	351	216.11	14.70	.001
	Male	321	92.30	9.61	
*G. m. centralis* locations	KNP1	340	379.27	19.40	.013
	KNP2	341	447.38	21.15	
	KSP	349	449.69	21.21	
	SNP	346	386.01	19.65	
*G. m. morsitans* locations	CMR	334	347.55	18.64	.001
	LVA	332	372.86	19.31	
	LZP	349	527.02	22.96	
	SLP	333	124.10	11.14	
	VNP	333	356.63	18.88	

Elevation, annual temperature, annual precipitation, and land surface temperature were observed to have a significant effect on *G. morsitans* wing CS (Table 3). The coefficients of the regression model indicated that land surface temperature had the largest per‐unit effect on CS whose net effect was a reduction in fly size.

**TABLE 3:** Effect of environmental variables on *G. morsitans* wing CS.

Variable	Coefficient	*p*‐Value
Elevation	0.0095	.001
Annual temperature	1.0223	.009
Isothermality	−0.1928	.280
Annual precipitation	0.0462	.001
Land surface temperature	−3.0115	.001
Per cent tree cover	0.16078	.459
Total		

### 3.4 Allometry

The covariation of wing shape with CS was found to be significant (Goodall's *F* Statistic = 93.62, *p < .001*). Allometry was observed to account for an estimated 12% of shape variation in *G. morsitans*.

### 3.5 Allometry‐free wing shape variation

**FIGURE 6:**
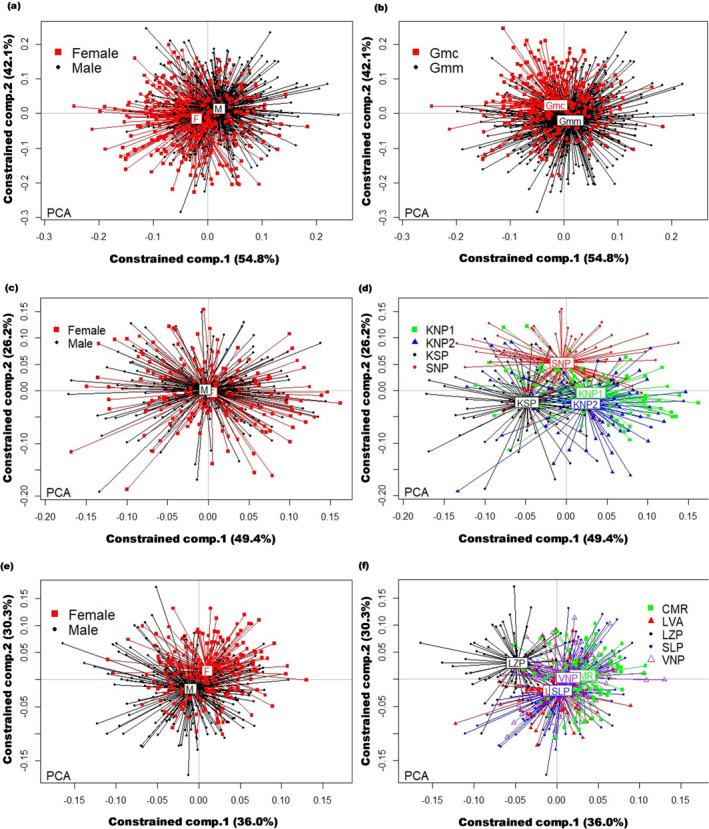
Redundancy Analysis (RDA) score plots from constrained PCA (PCA). (a) Score plot for the constrained PCA of allometry‐free wing shape of male and female *G. morsitans*. (b) Score plot for the constrained PCA of allometry‐free wing shape of *G. m. centralis* and *G. m. morsitans*. (c) Score plot for the constrained PCA of allometry‐free wing shape of male and female *G. m. centralis*. (d) Score plot for the constrained PCA of allometry‐free wing shape of *G. m. centralis* from different geographic locations. (e) Score plot for the constrained PCA of allometry‐free wing shape of male and females *G. m. morsitans*. (f) Score plot for the constrained PCA of allometry‐free wing shape of *G. m. morsitans* from different geographic locations.

Allometry‐free wing shape variation in *G. morsitans* was observed to be significantly different due to sex (*p = .001*), subspecies identity (*p = .001*), and the two‐way interaction between these factors (*p = .006*). Thus, the wing shape between male and female *G. morsitans* and between the subspecies *G. m. centralis* and *G. m. morsitans* was observed to be significantly different. Overall, sex and subspecies differences, as well as their interaction, accounted for 3.7% (*p = .001*) of the total allometry‐free wing shape variation observed in *G. morsitans*. As shown in Figure 6a,b, the constrained PCs one and two accounted for 54.8% and 42.1% of this variation, respectively. The first and second constrained PCs we able to discriminate the centroid shape clusters of male and female *G. morsitans* and those of *G. m. centralis* and *G. m. morsitans*, respectively (Figure 6a,b).

Sex differences (*p = .002*) and geographic origin (*p = .001*) were observed to significantly influence allometry‐free wing shape variation in *G. m. centralis*. However, the interaction between sex and geographic origin did not significantly affect allometry‐free wing shape (*p = .099*). Therefore, the allometry‐free wing shape in *G. m. centralis* was significantly different between males and females and between flies from different geographic locations. Overall, sex and location differences accounted for 10.3% (*p = .001*) of allometry‐free wing shape variation in *G. m. centralis*. The constrained PCs one and two explained 49.4 and 26.2% of this variation (Figure 6c,d). These constrained PCs did not discriminate *G. m. centralis* male and female wing shape clusters (Figure 6c). Pairwise comparisons of *G. m. centralis* allometry‐free wing shape by geographic origin showed that the wing shape of flies from KSP and SNP sites were significantly different from those from KNP1 and KNP2 and each other (Table 4). Wing‐shape of flies from KNP1 and KNP2 were not significantly different from each other (Table 4). The constrained PCs one and two discriminated *G. m. centralis* flies into three clusters (Figure 6d).

**TABLE 4:** Pairwise comparison of allometry‐free wing shape of *G. morsitans* from different locations.

	KNP1	KNP2	KSP
*G. m. centralis*			
KNP2	0.051	–	–
KSP	0.001	0.001	–
SNP	0.001	0.001	0.001

	CMR	LVA	LZP	SLP
*G. m. morsitans*				
LVA	0.001	–	–	–
LZP	0.001	0.001	–	–
SLP	0.001	0.584	0.001	–
VNP	0.029	0.001	0.001	0.016

For *G. m. morsitans*, sex differences (*p = .001*), geographic origin (*p = .001*), and the interaction between these two factors (*p = .001*) were observed to significantly affect allometry‐free shape variation. Therefore, size‐adjusted wing shape in *G. m. morsitans* was significantly different between males and females and between flies from different geographic locations. Sex and location differences as well as the interaction of these two factors accounted for 18.9% (*p = .001*) of the total allometry‐free shape variation in *G. m. morsitans*. An estimated 66.3% of this variation was explained by the constrained PCs one (36.0%) and two (30.3%) (Figure 6e,f). The constrained PCs one and two discriminated the wing shapes of male and female *G. m. morsitans* into two clusters (Figure 6e). Pairwise comparisons of *G. m. morsitans* allometry‐free wing shape by geographic origin showed that only flies from SLP and LVA had similar sized‐adjusted wing shapes (Table 4). The wing shape of flies from the other sites was significantly different (Table 4). Discrimination of *G. m. morsitans* size‐adjusted wing shape from different sampling sites on the constrained PCs one and two is shown in Figure 6f. Flies from LZP were well separated from all other sites.

Size‐adjusted wing shape of *G. morsitans* was observed to be significantly associated with elevation, annual temperature, isothermality, annual precipitation, land surface temperature, and per cent tree cover (Table 5). Collectively, these variables accounted for 10.7% of the observed variation in wing shape at the species level. Land surface temperature, annual precipitation, and isothermality contributed the most to this environmental variation (Table 5).

**TABLE 5:** Effect of environmental variables on *G. morsitans* wing shape.

Variable	*F*‐value	*p*‐Value	Per cent explained
Elevation	5.600	.001	0.71
Annual temperature	13.734	.001	0.81
Isothermality	17.656	.001	2.38
Annual precipitation	4.951	.001	2.33
Land surface temperature	17.965	.001	3.51
Per cent tree cover	12.185	.001	0.96
Total			10.70

**FIGURE 7:**
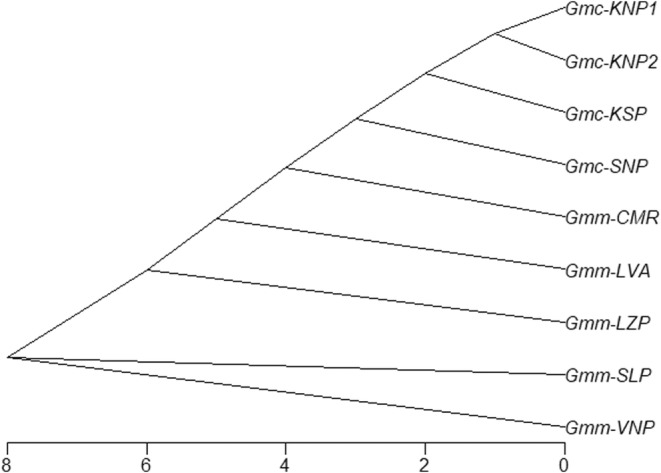
Cladogram of *G. morsitans* based on wing shape Procrustes distances. The figure indicates the divergence of *G. morsitans* wing shape based on subspecies and geographic origin.

The neighbour‐joining cladogram derived from the analysis of Procrustes distances indicated divergence of *G. morsitans* wing shape based on subspecies and geographic origin (Figure 7). The ancestral shape was observed among *G. m. morsitans* flies caught from the SLP and VNP sites. The wing shape of *G. m. centralis* appears to have diverged from that of *G. m. morsitans* caught from the CMR site (Figure 7). In *G. m. centralis*, flies from KNP1 and KNP2 were shown to be closely related while flies from KSP and SNP were divergent from this group and each other. For *G. m. morsitans*, flies from SLP and VNP were closely related while those from LVA, LZP, and CMR were divergent from this group and each other.

### 3.6 Isolation‐by‐distance

**FIGURE 8:**
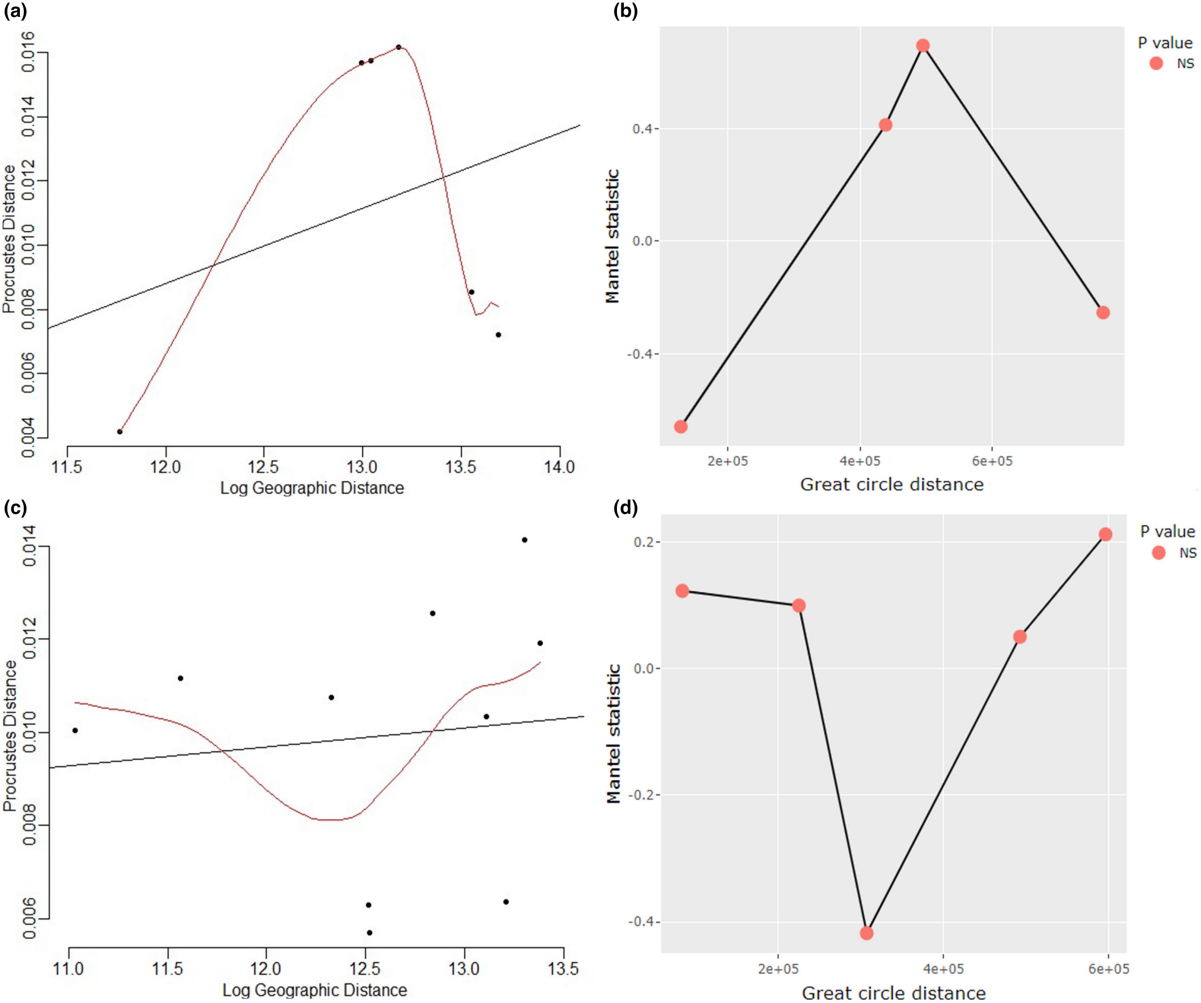
IBD plots. (a) Scatter plot of Procrustes distance vs. geographic distance for *G. m. centralis*. The plot suggests an increase in Procrustes distance with geographic distance. (b) Mantel correlogram of Procrustes and geographic distance for *G. m. centralis*. This plot indicates that Procrustes distance was uncorrelated with geographic distance. (c) Scatter plot of Procrustes distance vs. geographic distance for *G. m. morsitans*. The plot suggests no linear relationship between Procrustes and geographic distance. (d) Mantel correlogram of Procrustes and geographic distance for *G. m. morsitans*. This plot indicates that Procrustes distance was uncorrelated with geographic distance.

Scatter plots of Procrustes distance versus geographic distance suggested a linear relationship between the two variables for *G. m. centralis* (Figure 8a) but not for *G. m. morsitans* (Figure 8c). As shown in Table 6 and Figure 8b,d, all distance lags between sampling points did not show positive spatial autocorrelation. Therefore, the hypothesis that Procrustes distance increases with geographic distance in *G. m. centralis* and *G. m. morsitans* was rejected.

**TABLE 6:** Mantel correlogram analysis results for IBD tests.

Subspecies	Breaks of distance lag (m)	Mean distance (m)	Observed Mantel statistic	Expected Mantel statistic	*p*‐Value	Cardinal
*G. m. centralis*	0–219,831	129,194	−0.6591	0.4130	.20	1
	219,831–439,663	438,686	0.4130	0.4130	.61	1
	439,663–659,494	495,076	0.6948	0.0269	1.00	2
	659,494–879,325	767,470	−0.2537	−0.2537	1.00	1
*G. m. morsitans*	0–129,422	83,710	0.1229	0.0600	.63	2
	129,422–258,844	224,917	0.0997	0.0504	1.00	1
	258,844–388,267	306,886	−0.4177	−0.0151	.60	3
	388,267–517,689	492,314	0.0504	0.0997	1.00	1
	517,689–647,111	595,850	0.2121	0.0643	1.00	3

## 4 DISCUSSION

We employed a geometric morphometrics framework to elucidate the intraspecific phenotypic variability of the two subspecies of *G. morsitans* that occur in Zambia. Population‐level variability in CS and wing morphology can serve as a useful proxy for assessing the extent of divergence between conspecific populations (Ostwald et al., 2023) and may further provide preliminary data for the diagnosis of isolated populations (Dujardin, 2008). This information has important implications for the area‐wide integrated vector management (AW‐IVM) of *G. morsitans* in Zambia and further provides insights into the population differentiation status in its entire geographical range. Broadly, these results provide evidence for microevolutionary change in both CS and wing morphology in *G. m. centralis* and *G. m. morsitans* populations in Zambia.

Our results are consistent with the long‐held observation that size sexual dimorphism is well established in tsetse as female *G. morsitans* were found to be larger than male flies. The estimated CS difference between the two sexes (9 per cent) was similar to that reported by Hargrove et al. (2019), who found the wings of female *G. morsitans* to be 8 per cent longer than those of males. This observation provides further evidence that size studies based on wing measurements as described by Hargrove et al. (2019) and CS generated by geometric morphometric analysis, produce comparable results. Therefore, both measures are reliable estimators of mean wing size in *Glossina* spp.

This study has demonstrated that the mean wing size of *G. m. centralis* is larger than *G. m. morsitans*. It has been suggested that the size of tsetse is largely dependent on the nutritional state (Bursell, 1966) and temperature (Hargrove, 2001) experienced by the female. High temperatures exceeding 32°C result in tsetse entering cooler dark refuges such as rot holes in trees and antbear holes in the ground (Vale, 1971), a behaviour that reduces their metabolic rate but also reduces feeding opportunities (Lord et al., 2018). As such, female tsetse have reduced fat levels and produce progressively smaller pupae as temperature increases (English et al., 2016). Hargrove et al. (2018) showed that small pupae have lower fat reserves which results in the emergence of smaller‐sized adults. Thus, the smaller fly size of *G. m. morsitans* may be an adaptation to its occupation of a hotter environment than that of *G. m. centralis* as reported by Evison and Kathuria (1982) and Muyobela et al. (2023) and reaffirmed by our results. Location differences in mean wing size were observed in both subspecies' ranges and temperature is again implicated as the major source of fly size variation.

We postulate that the observed environmentally driven fly size variation between the two subspecies may be explained by the hypotheses of phenotypic plasticity and genetic assimilation (Dujardin, 2011). Phenotypic plasticity is defined as the occurrence of phenotypic variation of a single genotype interacting with different environments (Pigliucci et al., 2006). The observed within species differences in fly size are probably adaptive to the different ecotopes where *G. morsitans* occurs, with plastic responses facilitating the enlargement of its ecological range. Consequently, phenotypic plasticity may have aided *G. morsitans* to survive in both warm (*G. m. morsitans*) and cooler (*G. m. centralis*) environments within its range, by providing both small and large‐sized flies upon which natural selection has acted. It is conceivable that selection has resulted in fly size being genetically determined at the subspecies level through the process of genetic assimilation (Flatt, 2005), and has now become a heritable trait. Heritability for insect size has been demonstrated in *Anopheles* mosquitoes (Lehmann et al., 2006) and its transgenerational effects were shown in *G. f. fuscipes* (Mbewe et al., 2018).

Although fly size differences within the subspecies *G. m. morsitans* are known to occur (Bursell, 1966) and are reported in this study, it is unlikely that these within subspecies differences are heritable. This is because temperature variability within a subspecies range is expected to be less variable than across the subspecies range. Therefore, other factors that affect size variability such as host availability, the nutritional state of females, ovarian age, and capture month and year (Hargrove et al., 2019) are likely to be more important. Since these factors are highly variable within the subspecies range, they consequently do not exert selection in any specific direction. Fly size change driven by these factors is therefore unlikely to result in heritable change (Jirakanjanakit et al., 2007). As such, size is expected to be a poor discriminator of *G. morsitans* subspecies population structure.

Our results showed that allometry and environmental variability accounted for 11.6 and 10.7% of shape variation in *G. morsitans*. As such, we estimate that 77.7% of wing shape variation could be attributed to genetic effects, a finding in support of the suggestion by Patterson and Klingenberg (2007) that shape exhibits high genetic determinism. The low contribution of environmental variability to allometry‐free wing shape variation suggests that *G. morsitans* wing shape exhibits high environmental canalization, in agreement with results from other Diptera such as sand flies (Dujardin & Le Pont, 2004) and mosquitoes (Henry et al., 2010).

We found that wing shape in *G. morsitans* varies according to gender, subspecies, and geographic origin. The detection of allometric‐free shape sexual dimorphism indicates that the phenotypic expression of wing shape in this tsetse is sex‐specific. Shape sexual dimorphism has been reported in other Dipteran families such as Drosophilidae (Gilchrist et al., 2000) and Culicinae (Virginio et al., 2015). Gilchrist et al. (2000) suggest that the gender regulation of shape in the Diptera represents a developmental constraint during morphogenesis rather than adaptive change. Tsetse biology appears to support this view as female flies reproduce by adenotrophic viviparity (Vreysen et al., 2013) which may present a different aerial dynamic challenge to pregnant females compared to males, hence the need for female wings to be designed differently. Evidence of strong genetic determinism of wing‐shape sexual dimorphism in the Diptera has been presented by Cowley et al. (1986).

Subspecies wing shape variation in *G. morsitans* may be an adaptive trait as *G. m. centralis* and *G. m. morsitans* occur in different habitats with different aerodynamic conditions due to temperature differences. Temperature is known to significantly affect aerodynamic lift (Liu et al., 2015). As air temperature increases, its density decreases leading to a decrease in the amount of lift generated by the wings. Therefore, selection may be acting on the wing phenotypes of the two subspecies differently as *G. m. centralis* occupies a cooler environment than *G. m. morsitans*, thereby producing wing shapes aerodynamically suitable for their specific environments. Ray et al. (2016) showed that selective pressure resulting in large and small changes in the wing shape of *Drosophila* can lead to significant changes in key flight performance metrics, leading to improved manoeuvrability and agility.

Significant wing shape variation was also observed within the subspecies ranges of both *G. m. centralis* and *G. m. morsitans*. Since shape is known to be the output of polygenic genes (Patterson & Klingenberg, 2007), within subspecies shape variation may be due to local adaptation or random genetic drift. Within the *G. m. centralis* range, random genetic drift is perhaps the primary cause of the observed population structuring given that the KNP, KSP, and SNP populations are physically separated by large areas of unsuitable habitat (Muyobela et al., 2023) (Figure 1). Under such a spatial arrangement of populations, it is highly unlikely that gene flow will occur between these populations, and genetic drift is expected to quickly generate wing shape changes. Several field studies have implicated genetic drift as a source of shape variation among geographic isolates of conspecific populations (Camara et al., 2006; Dujardin, 2011; Henry et al., 2010; Kaba et al., 2012). Shape change due to genetic drift has also been demonstrated in the laboratory (Jirakanjanakit et al., 2007).

In the *G. m. morsitans* range, physical separation between sample locations does not occur (Figure 1). The observed population structuring at these locations could therefore be primarily due to local adaptation to the different environmental conditions between sample sites. A key prerequisite to local adaptation is restricted gene flow among population demes (Kawecki & Ebert, 2004). Limited gene flow within the *G. m. morsitans* range may be attributed to high habitat fidelity as the interchange of individuals between contiguous parts of the general population of this tsetse is reportedly limited (Bursell, 1966). Rapid adaptation of wing shape to different environmental conditions has also been observed in *Drosophila melanogaster* (Önder & Aksoy, 2022).

Our results show that *G. m. centralis* and *G. m. morsitans* populations in Zambia are highly structured and exhibit significant morphological divergence. This observation suggests that the implementation of tsetse population management technologies that target an entire isolated population may be technically feasible. However, to categorically designate populations as isolated, it is essential to estimate the number of migrants per generation or the levels of gene flow between them (Bouyer et al., 2007), and methods using morphometric variation are not suited for these tasks (Dujardin, 2008). Therefore, the results presented in this study only provide preliminary information justifying further investigation using molecular techniques to conclusively identify genetically isolated populations (Dujardin, 2008). This is particularly crucial in the *G. m. morsitans* range where physical separation of sample locations was not apparent. It should be noted, however, that some authors have suggested that results from geometric morphometric studies are comparable to those of molecular studies using microsatellite markers (Bouyer et al., 2007, 2010; Solano et al., 1999).

We conclude that *G. morsitans* populations in Zambia exhibit significant population‐level variation in body size and allometry‐free wing shape. This variation suggests high levels of population structuring that may be indicative of population isolation. Molecular studies to estimate the levels of gene flow between these populations and determine their levels of genetic isolation will be able to shed even more light on *G. morsitans* population structure in Zambia and possibly identify its underlying drivers.

## AUTHOR CONTRIBUTIONS

**Jackson Muyobela:** Conceptualization (equal); data curation (lead); formal analysis (equal); methodology (equal); visualization (lead); writing – original draft (lead); writing – review and editing (equal). **Christian W. W. Pirk:** Conceptualization (equal); data curation (supporting); formal analysis (equal); funding acquisition (equal); methodology (equal); supervision (supporting); writing – original draft (supporting); writing – review and editing (equal). **Abdullahi A. Yusuf:** Conceptualization (equal); data curation (supporting); formal analysis (supporting); methodology (equal); supervision (supporting); visualization (supporting); writing – original draft (supporting); writing – review and editing (equal). **Catherine L. Sole:** Conceptualization (equal); data curation (supporting); formal analysis (equal); funding acquisition (lead); investigation (lead); methodology (equal); project administration (lead); resources (lead); supervision (lead); visualization (equal); writing – original draft (supporting); writing – review and editing (equal).

## CONFLICT OF INTEREST STATEMENT

The authors declare that they have no competing interests.

## Supporting information


Data S1

## Data Availability

All relevant data are provided with the manuscript and its Supporting Information files.
